# Porphyrin-Based Organoplatinum(II) Metallacycles With Enhanced Photooxidization Reactivity

**DOI:** 10.3389/fchem.2020.00262

**Published:** 2020-04-28

**Authors:** Lintao Wu, Chun Han, Zhijun Wang, Xi Wu, Feng Su, Mengyao Li, Qingyang Zhang, Xiaobi Jing

**Affiliations:** ^1^Department of Chemistry, Changzhi University, Changzhi, China; ^2^State Key Laboratory of Bioactive Substances and Function of Natural Medicine, Institute of Materia Medica, Chinese Academy of Medical Sciences and Peking Union Medical College, Beijing, China; ^3^School of Chemistry and Chemical Engineering, Yangzhou University, Yangzhou, China

**Keywords:** self-assembly, macrocycle, porphyrin, photooxidization, coordination

## Abstract

In recent years, metal coordination macrocycles have obtained great interests due to the fact that they combined the rich host-guest properties of macro-cyclic hosts and the unique optical properties of the organic ligands. In this work, we constructed two porphyrin-based organoplatinum(II) metallacycles (**MC1** and **MC2**) through coordination-driven self-assembly. ^1^H NMR, ^31^P NMR, and HRMS technologies were used to characterize the structures of **MC1** and **MC2**. Interestingly, **MC1** and **MC2** can be used as catalysts for photooxidization under light irradiation with higher efficiency compared with the porphyrin ligand only. We hope that the coordination-driven self-assembly strategy can provide an efficient method to construct photo-active materials.

## Introduction

Macrocyclic host compounds, mainly including crown ethers (Zhu et al., 2012; Liu et al., 2017), cyclodextrins (Lai et al., 2017; Li et al., 2019), calixarenes (Kim et al., 2012; Nimse and Kim, 2013), cucurbiturils (Kim et al., 2007; Barrow et al., 2015), and pillararenes (Xue et al., 2012; Ogoshi et al., 2016; Yao et al., 2017; Chen J. et al., 2019), are the foundation of the development of supramolecular chemistry (Dong et al., 2014; Sun et al., 2018; Gao L. et al., 2019; Xiao et al., 2019). During the past two decades, the syntheses, host–guest properties, and applications of macrocycles have been widely investigated (Chen Y. et al., 2019; Wu and Yang, 2019). Among various macrocycles, discrete organoplatinum(II) metallacycles, which was fabricated by a new valuable strategy called “coordination-driven self-assembly,” attracted great interests from both chemists and materials scientists (Gao S. et al., 2019; Zhang et al., 2019). A remarkable advantage of the “coordination-driven self-assembly” is that two-dimensional metallacycles or three-dimensional metallacages can be easily obtained by the formation of metal–ligand bonds between metal acceptors and organic donors when combining simple building blocks (Wang et al., 2019a; Yan et al., 2019). Up to now, discrete organoplatinum(II) metallacycles have been investigated a lot and widely applied in many areas, such as fluorescent detection, homogeneous catalysis, functional materials, bioengineering, photodynamic therapy, and so on (Cai et al., 2020; Qin et al., 2019).

Porphyrin derivatives, which contain a large π-conjugated aromatic structure, are a class of famous photo-activities (Liang et al., 2011; Ou et al., 2019; Wang et al., 2019b). Porphyrins usually have very intense absorption bands in the UV–visible region. However, due to the strong π-π stacking between the aromatic systems, porphyrins are easily aggregated in solvents, especially in aqueous solution (Zou et al., 2017). Commonly, porphyrins aggregate more seriously as the concentration increased. This aggregation phenomenon greatly decreases the efficiency of porphyrins to generate ^1^O_2_ and therefore restrained their potentially wide applications (Zhou et al., 2019). To address the aggregation of porphyrins in water, chemistry and materials scientists usually introduce a large substituent onto the platform of the porphyrin core (Slater et al., 2015). However, these chemical synthesis and purification processes have some other disadvantages, such as being time-consuming, tedious, and with higher costs of preparation.

**Scheme 1:**
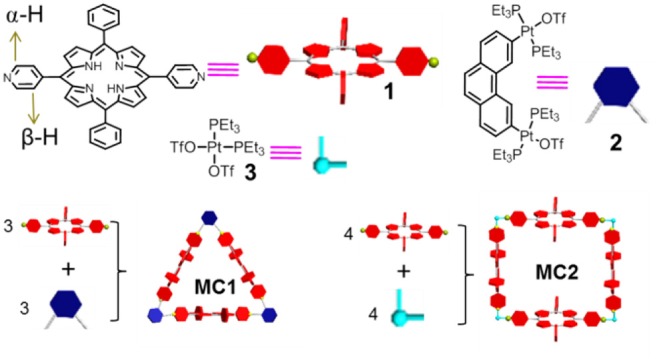
Chemical structures and schematic diagram of *p*-bipyridine-modified porphyrin **1**, organoplatinum(II) **2** and **3**, and metallacycles **MC1** and **MC2**.

Herein we designed and synthesized two new metallacycles (**MC1** and **MC2**) with *p*-bipyridine-modified porphyrin (Scheme S1, Scheme 1) as organic donor and organoplatinum(II) (**2** or **3**) as the metal acceptor (Scheme 1). The weak metal–ligand bonds will prevent the π-π stacking of the conjugated aromatic porphyrin units, thus improving the efficiency of generating ^1^O_2_ under irradiation. Interestingly, compared with ligand **1** (Figure S1), the resultant metallacycle **MC1** or **MC2** can be used as catalyst for photo-oxidizing phenols much more efficiently.

## Materials and Methods

### Synthesis of Metallacycles MC1 and MC2

**Figure 1:**
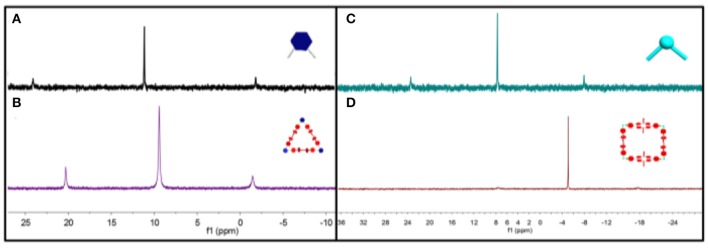
^31^P{^1^H} NMR spectra (room temperature, 121.4 MHz) of **(A)** 60° acceptor **2**, **(B)** metallacycle **MC1**, **(C)** 90°acceptor **3**, and **(D)** metallacycle **MC2** in acetone.

Ligand **1** and organoplatinum(II) **2** (Figure 1A) and **3** (Figure 1C) were prepared according to a previous report (Grishagin et al., 2014). In a 1:1 molar ratio, bipyridylporphyrin **1** (1.85 mg, 3.00 μmol) and 60°Pt (II) acceptor **2** (4.01 mg, 3. 00 μmol) were placed in a 2-ml vial, followed by addition of acetone (1 ml). After stirring overnight at 50°C, the mixture was filtered to remove insoluble materials (Scheme S2). Then, the solvent was removed by N_2_ flow to about 0.2 ml, and **MC1** was obtained by the addition of diethyl ether (5.22 mg, 89%). **MC2** was prepared by the same method (Scheme S3).

#### MC1

Purple solid, 89%. ^1^H NMR (400 MHz, CD_3_COCD_3_) δ (ppm): 10.17 (d, 4H), 9.21 (s, 2H), 8.92–8.90 (m, 10H), 8.27–8.25 (m, 8H), 7.89–7.85 (m, 12H), 2.47–2.43 (m, 24H), 1.70–1.62 (m, 36H). ^31^P {^1^H} NMR (acetone, room temperature, 121.4 MHz) δ = 9.53 (^195^Pt satellites, ^1^*J*_Pt−P_ = 2,662 Hz). HR-ESI-MS: calculated for C_203_H_288_F_9_N_18_O_9_P_12_Pt_6_S_3_ ([M – 3 OTf]^3+^): 1,646.78, found: 1,646.77.

#### MC2

Purple solid, 87%. ^1^H NMR (400 MHz, CD_3_COCD_3_) δ (ppm): 9.73 (s, 1H), 9.61 (s, 1H), 9.06 (s, 2H), 8.90 (s, 4H), 8.67–8.65 (m, 2H), 8.29–8.19 (m, 4H), 7.86–7.74 (m, 12H), 1.83–1.81 (m, 24H), 1.49–1.41 (m, 36H). ^31^P {^1^H} NMR (acetone, room temperature, 121.4 MHz) δ = −5.04 ppm (^195^Pt satellites, ^1^*J*_Pt−P_ = 3,156 Hz). HR-ESI-MS: calculated for C_244_H_280_F_12_N_24_O_20_P_8_Pt_4_S_4_ ([M + 8 CH_3_COCH_3_ – 4 OTf]^4+^): 1,316.97, found: 1,316.92.

### Materials

All reagents and solvents were commercially available in analytical grade and used as received. Further purification and drying by standard methods were employed and these were distilled prior to use when necessary. Deuterated solvents were purchased from Cambridge Isotope Laboratory (Andover, MA, USA). All evaporations of organic solvents were carried out with a rotary evaporator in conjunction with a water aspirator. Melting point measurements were taken on a hot-plate microscope apparatus and are uncorrected. ^1^H and ^13^C NMR spectra were recorded with an Aviance III 400 MHz or 600 MHz liquid-state NMR spectrometer. ^31^P{^1^H} NMR chemical shifts are referenced to an external unlocked sample of 85% H_3_PO_4_ (δ 0.0). Mass spectra were recorded on a Micromass Quattro II triple–quadrupole mass spectrometer using electrospray ionization with a MassLynx operating system. UV–vis spectra were recorded on a Hitachi F-7000 fluorescence spectrophotometer.

## Results and Discussion

### NMR Studies

The formation of discrete organoplatinum(II) metallacycles **MC1** and **MC2** were characterized by multinuclear NMR (^31^P and ^1^H) analysis. The ^31^P {^1^H} NMR spectra of **MC1** and **MC2** showed a sharp singlet with concomitant ^195^Pt satellites at 9.53 ppm for **MC1** and at −5.04 ppm for **MC2** (Figures 1B,D) corresponding to a single phosphorous environment, indicating the formation of discrete and symmetric metallacycles (Wei et al., 2014).

**Figure 2:**
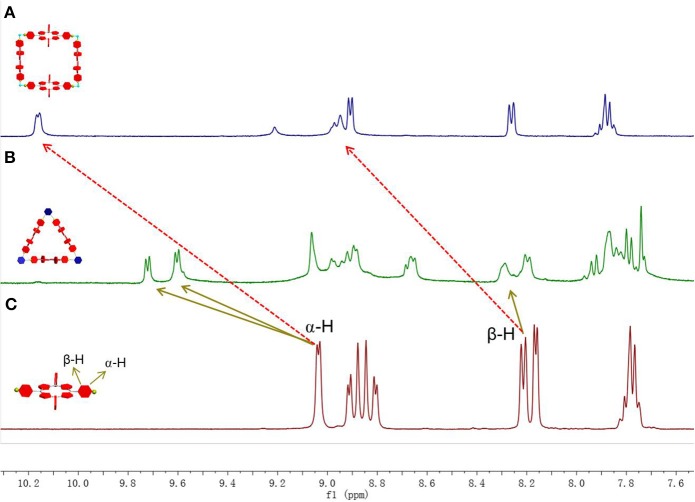
^1^H NMR spectra (CD_3_COCD_3_, room temperature) of **(A)** bipyridylporphyrin **1**, **(B)** metallacycle **MC1**, and **(C)** metallacycle **MC2**.

At the same time, downshifts were observed for β-pyridyl hydrogen in ^1^H NMR spectra. As shown in Figure 2, β-pyridyl hydrogen changed from 9.04 to 9.51 and 9.72 ppm in **MC1** and from 9.04 to10.21 ppm in **MC2**. β-pyridyl hydrogen also showed a downfield chemical shift. These chemical shift changes in ^1^H NMR spectra are similar with the previous analogous organoplatinum(II) system, indicating the formation of discrete metallacycles (Yao et al., 2018).

### Electrospray Ionization Time of Flight Mass Spectrometry Studies

**Figure 3:**
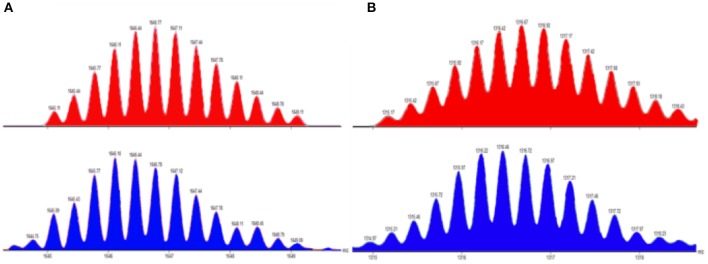
Experimental (blue) and calculated (red) ESI-TOF-MS spectra of **(A)** [M – 3OTf]^3+^ and **(B)** [M + 8 CH_3_COCH_3__Â_ 4OTf]^4+^.

Electrospray ionization time of flight mass spectrometry (ESI-TOF-MS) provided further evidence for the stoichiometry formation of discrete metallacycles **MC1** and **MC2**. In the mass spectrum of **MC1**, the peak at m/z = 1,646.77 is consistent with an intact [M – 3OTf]^3+^ charge state, which supported a [3 + 3] metallacycle (Figure 3A). Similarly, for metallacycle **MC2**, the peak at m/z = 1,316.92 is consistent with an intact [M + 8 CH_3_COCH_3_^−^ 4OTf]^4+^ charge state, which is expected only for a [4 + 4] metallacycle (Figure 3B). All the evidence from ^1^H NMR, ^31^P NMR, and ESI-TOF-MS confirmed the formation of a discrete structure as the sole assembly product.

### Photooxidization Studies

**Figure 4:**
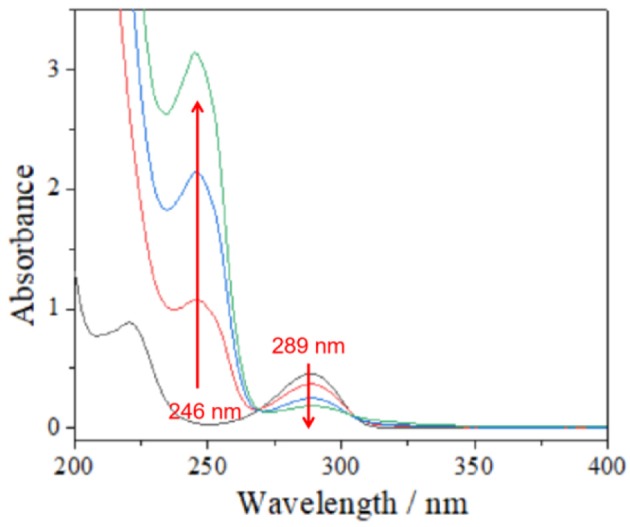
UV–vis spectra of quinol solution with **MC1** upon light irradiation at 500 nm with a xenon lamp.

As we all know, porphyrins have the ability to generate ^1^O_2_ due to the fact that they could be excited into ^3^O_2_ state under irradiation and the energy transfer process is accompanied with molecular O_2_. However, due to the strong π-π interactions, most porphyrins applied as photosensitizers are easily aggregated in aqueous solution (Figures S4, S5). This aggregation will greatly restrain the ability of the porphyrins to generate reactive oxygen species. For our obtained metallacycles **MC1** and **MC2**, the coordination bonds will decrease the self-quenching of the excited states and improve the photooxidization efficiency. Therefore, metallacycles **MC1** and **MC2** can be used as an expected catalyst for the photoreaction mediated by ^1^O_2_. Herein quinol was selected as a model substrate for detecting the reactivity, and UV–vis spectroscopy was used to monitor the process. As shown in Figure 4, after 20 ml of aqueous solution of quinol (10^−2^ mmol L^−1^) was irradiated by a LED lamp (500 nm) under air with **MC1** (5 mg) as catalyst, the absorption band of the phenyl moiety in quinol in 289 nm gradually decreased, and 65% of quinol was consumed after irradiation for 60 min (Figure 4). As expected, **MC2** has a similar catalytic efficiency with **MC1** (Figure S2). However, in the control experiments using the ligand **1** as catalyst instead of **MC1**, only 8% of quinol was reacted after irradiation at 500 for 60 min under the same conditions (Figure S2). Importantly, the investigation for the recyclability of **MC1** showed that they could be recovered by simple filtration and reused without significant loss of catalytic activity (yield loss within 5% for six cycles, Figure S3).

## Conclusions

In this paper, we synthesized two metallacycles, **MC1** and **MC2**, with *p*-bipyridines modified porphyrin as the ligands through coordination-driven self-assembly. Then, the obtained metallacycles were characterized by ^31^P NMR, ^1^H NMR, and ESI-TOF-MS methods. Furthermore, the metallacycles **MC1** and **MC2** can be used as an expected catalyst for the photoreaction mediated by ^1^O_2_ due to the coordination bonds that will decrease the self-quenching of the excited states of porphyrin units and improve the photooxidization efficiency. Our next study will focus on the application of our metallacycles in photodynamic therapy.

## Data Availability Statement

All datasets generated for this study are included in the article/Supplementary Material.

## Author Contributions

LW, CH, and ZW prepared the ligands. LW, XW, and FS constructed the metallacycles. ML and QZ did the photooxidization. LW and XJ analyzed the data. LW, QZ, and XJ wrote the paper.

## Conflict of Interest

The authors declare that the research was conducted in the absence of any commercial or financial relationships that could be construed as a potential conflict of interest. The reviewer ML declared a shared affiliation, with no collaboration, with one of the authors QZ to the handling editor at time of review.
