# Supplementary material for: Porphyrin-Based Organoplatinum(II) Metallacycles With Enhanced Photooxidization Reactivity
Source: Front Chem. 2020 Apr 28;8:262. doi: 10.3389/fchem.2020.00262 (PMC7199493; doi:10.3389/fchem.2020.00262)
Supplement: Supplementary file 1 [file Data_Sheet_1.pdf]

# Porphyrin based organoplatinum(II) metallacycles with enhanced photooxidization reactivity

Lintao Wu[1], Chun Han[1], Zhijun Wang[1], Xi Wu[1], Feng Su[1], Mengyao Li[1], Qingyang Zhang[**3], Xiaobi Jing[*2]

[1]*Department of Chemistry, Changzhi University; Changzhi, Shanxi, 046000, China*

[2]*School of Chemistry and Chemical Engineering, Yangzhou University, Yangzhou, Jiangsu 225002, China.*

[3]*State Key Laboratory of Bioactive Substances and Function of Natural Medicine, Institute of Materia Medica, Chinese Academy of Medical Sciences & Peking Union Medical College, Xian Nong Tan Street, Beijing, 100050, China.*

## 1. Synthesis of ligand **1**

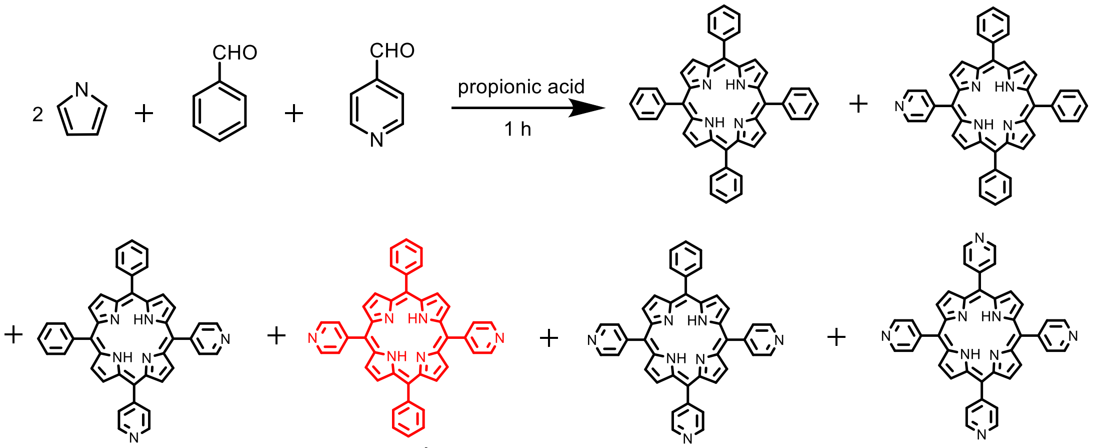


Scheme S1. Synthetic route to ligand **1**.

Ligand **1** was synthesized according to a modified procedure[S1]. Pyrrole (7.0 mL, 100 mmol), benzaldehyde (5.0 mL, 50 mmol), and 4-pyridinecarboxaldehyde (5.0 mL, 50 mmol) were refluxed in 250 mL of 99% propionic acid for 1 h. The reaction mixture was then cooled and allowed to stand overnight. Filtration and methanol washing afforded 4.15 g (26% yield) of a purple crystalline product. The product was analyzed by silica gel thin layer chromatography and found to be a mixture of the six possible porphyrin isomers, ligand **1** was the fourth one and has a 8% yield. The $^1$H NMR spectrum of **1** is shown in Figure S1. $^1$H NMR ($CDCl_3$, room temperature, 400 MHz) $\delta$ (ppm): 9.04 (4 H, d, 2,6-pyridyl), 8.91 (8 H, m, pyrrole $\beta$), 8.21 (8 H, m. *o*-phenyl. and 3,5-pyridyl), 7.78 (6 H, m, *m*- and *p*-phenyl).

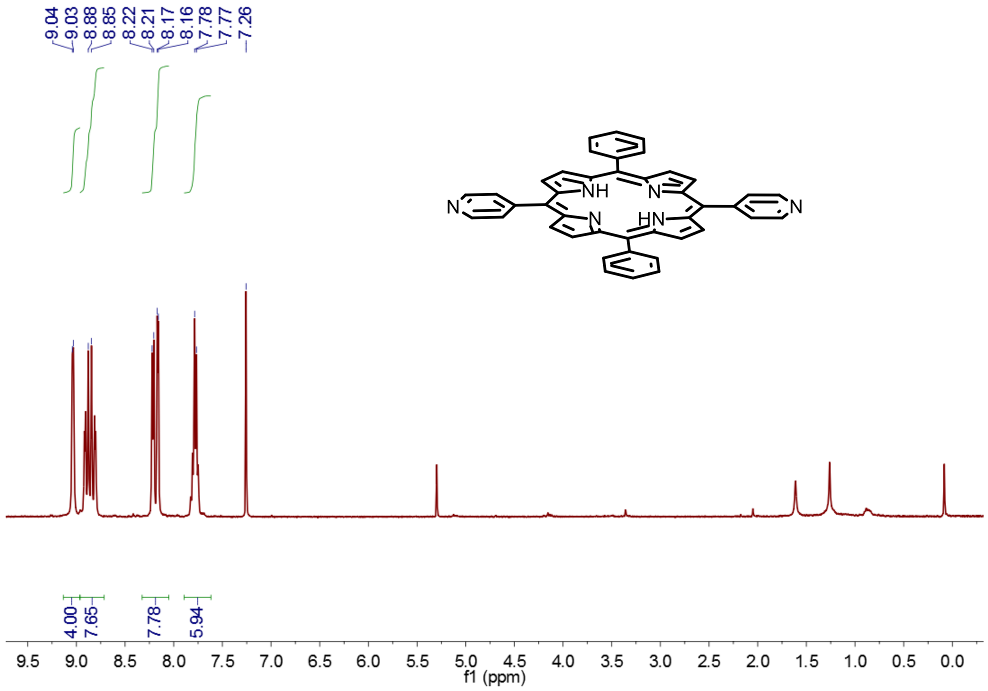


**Fig. S1.** $^1$H NMR spectrum ($CDCl_3$, room temperature, 400 MHz) of ligand **1**.

2. *Synthesis of metallacycle **MC1***

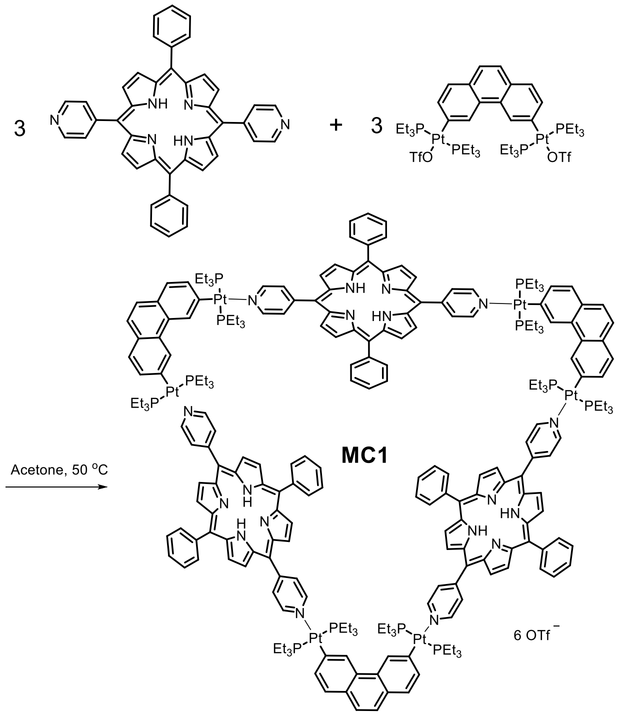


Scheme 2. Synthetic route to **MC1**.

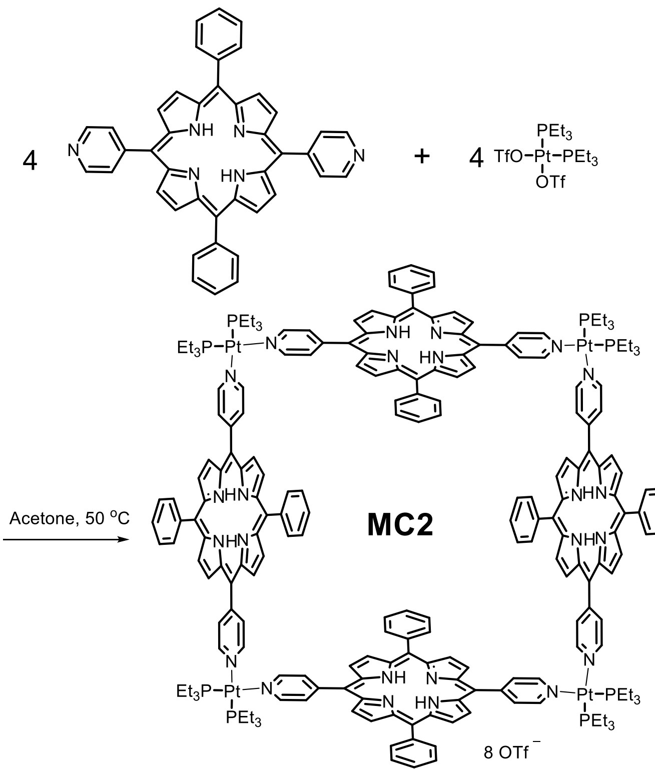


Scheme 3. Synthetic route to **MC2**.

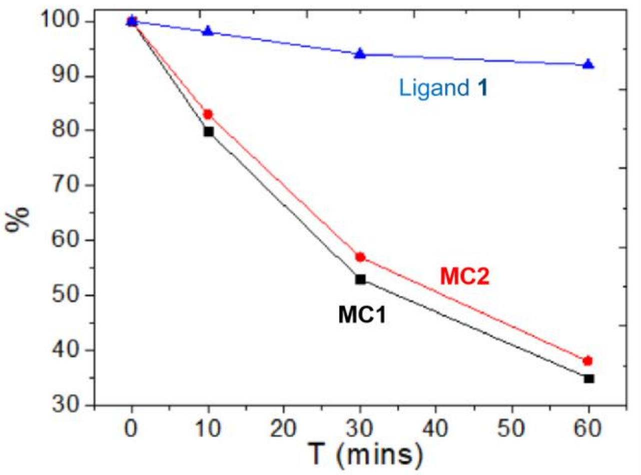


**Fig S2.** Plots of quinol content versus irradiation time for **MC1**, **MC2** and ligand **1** by monitoring the absorbance intensity at 289 nm.

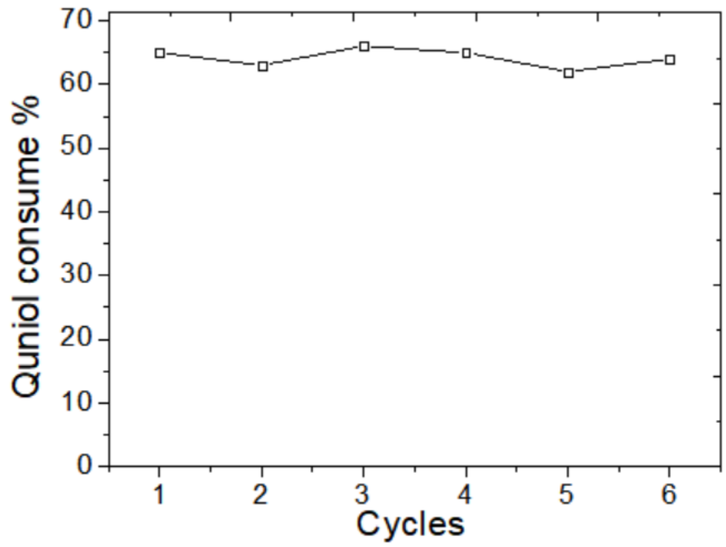


**Fig S3.** Quinol consume versus **MC1** reuse times when irradiation 60 min.

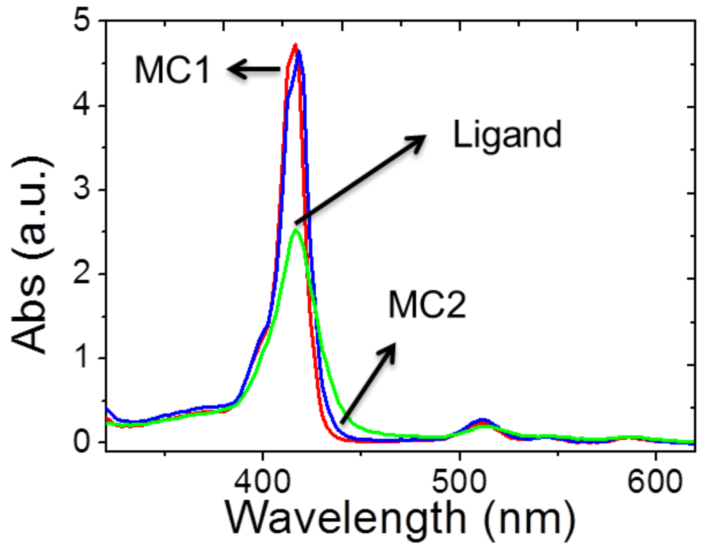


**Fig. S4.** UV–vis absorption spectra of the ligand, **MC1** and **MC2** in DMF.

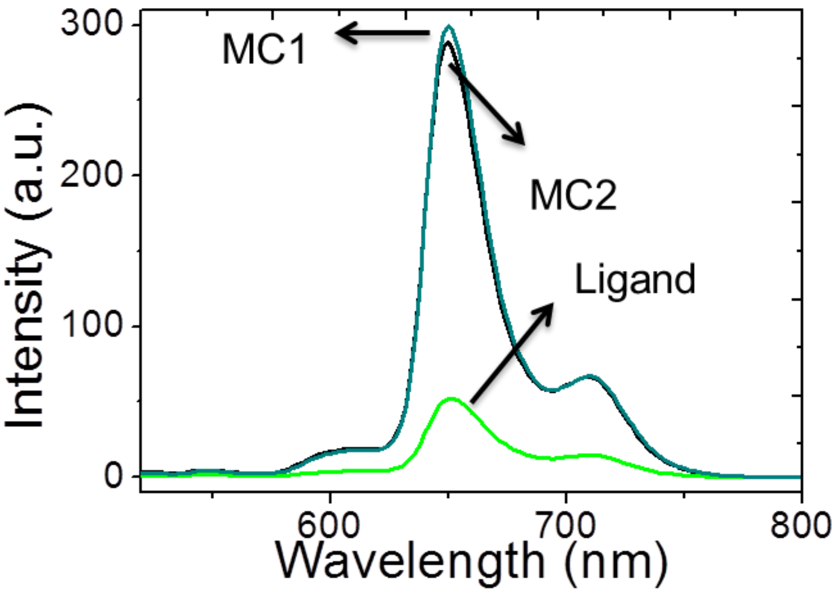


**Fig. S5.** Fluorescence emission of the ligand, **MC1** and **MC2** in water.
